# Co‐Creation in Research: Further Reflections From the ‘Co‐Creating Safe Spaces’ Project

**DOI:** 10.1111/hex.70103

**Published:** 2024-11-15

**Authors:** Scott J. Fitzpatrick, Heather Lamb, Erin Oldman, Melanie Giugni, Cassandra Chakouch, Alyssa R. Morse, Amelia Gulliver, Erin Stewart, Stride Safe Space and Safe Haven Team, Helen T. Oni, Benn Miller, Bronwen Edwards, Kelly Stewart, Vida Bliokas, Louise A. Ellis, Fiona Shand, Alison L. Calear, Michelle Banfield

**Affiliations:** ^1^ Centre for Mental Health Research The Australian National University Canberra Australian Capital Territory Australia; ^2^ Roses in the Ocean Brisbane Queensland Australia; ^3^ Black Dog Institute University of New South Wales Sydney New South Wales Australia; ^4^ Independent Researcher Canberra Australian Capital Territory Australia; ^5^ Sonder Adelaide South Australia Australia; ^6^ South Western Sydney Local Health District Sydney New South Wales Australia; ^7^ Northern Adelaide Local Health Network Adelaide South Australia Australia; ^8^ School of Psychology University of Wollongong Wollongong New South Wales Australia; ^9^ Australian Institute of Health Innovation Macquarie University Sydney New South Wales Australia; ^10^ ALIVE National Centre for Mental Health Research Translation Melbourne Victoria Australia

**Keywords:** co‐creation, co‐design, knowledge production, participatory research, patient and public involvement, safe spaces, suicide prevention

## Abstract

**Background:**

Applied research using co‐creation methods is rarely described or evaluated in detail. Practical evidence of co‐creation processes and collaboration effectiveness is needed to better understand its complex and dynamic nature.

**Methods:**

Using a case study design and survey method, we assessed processes of co‐implementation and co‐evaluation grounded in our own experiences from the Co‐Creating Safe Spaces project. We examine these in the context of a published systematic framework designed to improve clarity about co‐creation processes and report on how co‐creation was experienced by collaborative partners.

**Results:**

Our study showed the interconnectedness between co‐implementation and co‐evaluation processes and the importance of aligning research with program processes to ensure it is responsive to emergent local needs and problems. Given relatively low levels of researcher embeddedness across sites, service champions played a pivotal role in data collection. Survey findings indicated strong support for a healthy collaboration with some concerns expressed over individual partner's areas of responsibility and ability to deliver on commitments.

**Conclusion:**

Co‐creation can be a very robust approach to translational research but is a complex endeavour. Ongoing reflexivity and attention to relational aspects support genuine collaboration and provide a foundation for addressing challenges.

**Patient or Public Contribution:**

People with lived experience of emotional distress and/or suicidal crisis, including researchers from both academic and non‐research backgrounds, service managers, peer workers, carers and advocates, were involved in this research and authored this paper.

## 1 Background

Research co‐creation is a widely used term for describing collaboration between researchers and research end‐users to ensure optimal design, implementation and evaluation of initiatives involving multiple stakeholders [1]. Referring to collaborative research practices and related activities that support research engagement, translation and impact, co‐creation focuses on developing solutions to priority problems or issues [2, 3]. Despite the growing usage of the term, co‐creation research lacks definitional clarity and is applied differently across different settings and disciplines [4]. Improved clarity of terminology can be helpful for understanding involvement and engagement across the research cycle and related activities, helping to differentiate co‐created research from other types of research [4]. To this end, Pearce et al. [4] identified four primary categories of collaborative processes in the literature on co‐creation: (1) co‐ideation, (2) co‐design, (3) co‐implementation and (4) co‐evaluation. Key to this framework and the translational research models it seeks to advance is an understanding that co‐creation involves a simultaneous focus on the ideation, planning, implementation and evaluation of new services and programs alongside the generation of new knowledge, with an equitable collaboration between stakeholders an essential component [4]. This is particularly relevant in the field of suicide prevention where incomplete and insufficient knowledge of ‘what works’ necessitates the generation of new knowledge in parallel with the design, delivery and evaluation of new services and programs.

In the absence of a universally accepted definition of co‐creation, we aim to explore in this article the utility of Pearce and colleagues' [4] framework in light of our co‐created research project, ‘Co‐Creating Safe Spaces’. Specifically, using an instrumental case study design we aim to describe and develop two of the proposed constructs: co‐implementation and co‐evaluation, having already explored co‐ideation and co‐design processes in a previous work [5]. To extend the framework's utility, and, in particular, the construct of co‐evaluation, we seek to operationalise it by employing a theory‐driven survey tool to co‐evaluate the ‘health’ of our collaborative.

## 2 Methodology and Methods

### 2.1 Study Setting

Co‐Creating Safe Spaces was a national multisite study of six safe spaces, which are nonclinical peer‐led services for people experiencing suicidal crisis or distress. It used a mixed‐methods, co‐created study design to facilitate both a quantitative understanding of access, use, satisfaction, distress and cost‐effectiveness; and a qualitative understanding of service implementation, experience, feasibility, community awareness, acceptability and the fidelity of the models to service co‐design (a process separate to the research project described here). For details on the full study, please see the published research protocol [6].

Co‐Creating Safe Spaces involved a core team of lived experience representatives, health and community service managers and academic researchers, the majority of whom identified as having a lived experience of suicide, either through their personal experience of suicidal crisis or distress, or as supporting a family member through this experience. The project was planned and funded to embed co‐creation into all aspects and stages of research from conception through to dissemination. The core team comprised between 20 and 30 people, with numbers fluctuating at times due to personnel changes within several organisations. The core team met approximately 2‐monthly from November 2021, with members of safe space steering committees from three services and Roses in the Ocean, a leading Australian lived experience of suicide organisation, assembling before this date to collaborate on study conceptualisation, design and grant preparation, as well as the approach to co‐creation.

In addition to regular meetings that provided progress updates and problem solving of practical barriers, the core team was involved in several co‐creation activities including (i) a workshop held in November 2021 to establish shared values and principles for working together as a group; (ii) an ideas generation workshop for safe space staff to provide input on outcome measures for safe space guests and staff; (iii) an online survey for health services and safe space site partners to provide input on outcome measures and data collection methods for safe space guests, staff and for the health system(s) in which safe spaces were being implemented; (iv) a series of online and face‐to‐face meetings with staff at individual safe space sites to discuss final outcome measures and procedures for implementing data collection processes and (v) an analysis plan and strategic integration workshop on how to best integrate data to report on study findings and to allocate primary responsibilities for analysis and write‐up. In addition, all core team members were invited to co‐author project outputs and were co‐authors on the protocol [6]. At the time of writing the current paper, research, service and lived experience representative partners were collaborating on data analysis, interpretation and writing of papers detailing individual sub‐study findings.

### 2.2 Methodology

To gain a broader understanding of co‐creation and the utility of Pearce and colleagues [4] framework for understanding collaborative processes of co‐implementation and co‐evaluation, we undertook an instrumental case study grounded in our own experience of the Co‐Creating Safe Spaces project. Case studies are used to generate in‐depth, context‐dependent knowledge in a natural setting to increase understanding of complex and broad topics or phenomena [7, 8]. In an instrumental case study, a particular case is used to gain insight into an issue, for example, how co‐creation processes exist as an exemplar within a particular project [9]. From this perspective, the case is important due to its contexts and activities, yet its primary value is that it provides knowledge of the area of interest and an opportunity to learn from it. Given the wider social and institutional contexts that shaped Co‐Creating Safe Spaces, the authors adopted a critical reflective approach [10]. Critical reflection is a process of analysing individual practice within local organisational contexts to make sense of experience and implicit ways of working [11]. Adopting this approach, we sought to document and reflect upon processes of co‐implementation, co‐evaluation and the resultant challenges to provide insights into participation and collaboration within our multisite co‐created research project.

### 2.3 Survey Design

An important feature of the project was to use process evaluation tools to evaluate our ways of working to ensure that we were true to the principles agreed to at the outset [6]. These included trust, inclusion, choice, transparency, safety, lived‐experience‐led and valuing each person's experience and expertise [5]. To do this, core team members participated in an online survey designed to co‐evaluate ‘collaboration health’ across multiple domains.

Several different evaluation tools for identifying and measuring broad aspects of collaboration exist. The Collaborative Health Assessment Tool (CHAT) is a theory‐driven tool for measuring the ‘health’ of collaborative practices [12]. Viewed as a relational process in which stakeholders equitably participate in setting the agenda and developing solutions together, collaboration is an important facilitator of co‐created research. The CHAT was developed to recognise the complex and dynamic nature of collaborative systems and is conceptualised around two dimensions: (1) structure, defined as the ‘administrative design characteristics of the collaborative that guide collective action’ and (2) process, defined as ‘the relational dimensions that define members' interactions with each other and with their environment, and that enable collaborative relationships' [13]. The Trust and Empowerment Inventory is designed to assess community involvement in areas relating to trust, respect, fairness, progress and effectiveness [14].

Our survey comprised 12 items: nine from the CHAT [12], one from the augmented CHAT [13] and two from the Trust and Empowerment Inventory [14]. The pool of 67 items was independently assessed and then discussed by the project team (seven members) to identify the items most relevant for the Co‐Creating Safe Spaces project, balancing depth of understanding with length of the survey. We aimed for a survey that could be completed in approximately 5–10 min and that could be accessed online via mobile devices. Items from the CHAT and augmented CHAT were used to measure collaboration in terms of structure (shared goal, shared resources, shared authority, shared accountability) and process (whole system engagement, communication flows, holding environment) [13]. To assess areas not well‐covered by the CHAT items, additional items from the Trust and Empowerment Inventory were used to assess engagement and decision‐making power [14]. All items were scored using a 5‐point Likert scale, ranging from 1 (strongly disagree) to 5 (strongly agree). The survey was created in Qualtrics.

### 2.4 Data Collection

To co‐evaluate collaborative practice, members of the Co‐Creating Safe Spaces core team were invited to complete the online survey. Of the 28 invitations sent, 20 surveys were completed (71% response rate). The survey was anonymous and collected only limited identifying information on lived experience and broad project role (research partner or service/lived experience partner). As the study focused on assessing collaborative practices within a large team who were all involved as co‐researchers rather than participants, human research ethics approval was not required.

### 2.5 Data Analysis

We used descriptive statistics with results reported as proportions. Results were collated and analysed by the project team (6 members) to interpret the survey data within broader social and institutional contexts. Findings from the survey analysis were then synthesised by the first author (S.F.) and presented to co‐authors, with reflection and feedback elicited in meetings and during the preparation and approval of the final manuscript.

## 3 Findings

We begin by documenting co‐implementation and co‐evaluation processes as they occurred within the Co‐Creating Safe Spaces project. We then report results from the online survey designed to assess collaborative practice within the Co‐Creating Safe Spaces project across multiple domains.

### 3.1 Co‐Implementation: The Alignment of Research and Program Processes

For Pearce et al. [4], co‐implementation refers to the alignment of the co‐designed program, policy or procedure with the research protocol. The focus, therefore, is primarily on the delivery of services and programs, with procedures for data collection being implemented to evaluate issues such as staff training and development, as well as barriers to, and enablers for, change. With co‐design and implementation of the six safe spaces occurring separately to the co‐created research project, co‐implementation in Co‐Creating Safe Spaces focused primarily on embedding data collection into routine continuous practice to enable continual feedback on indicators and outcomes of importance [15]. This was guided by a collaborative Multi‐Institutional Agreement (MIA) with clear objectives and sharing of roles and responsibilities. Although membership of the core team changed over the duration of the project as a result of personnel changes within organisations, a strong productive partnership was maintained between academic researchers, health and community service managers and lived experience representatives. These linkages were viewed as critical to ensuring that research was integrated within implementation processes and that findings could be considered in ongoing program improvement and planning.

Data collection for those using the safe spaces (guests) was embedded in the routine continuous practices of the safe spaces in the form of administrative data (reason for visit, distress level, support and information needs). These data were used to monitor service usage in the early stages of implementation and helped some sites to leverage additional funding to extend opening hours and increase staffing as a result of the high demand reported. Administrative data were shared with the research team for inclusion in the project.

Additional data collection tools for guests, co‐designed specifically for the project, were co‐implemented with services on a site‐by‐site basis. This resulted in a number of challenges. These centred mainly around the heavy workloads of peer staff working within the safe spaces and the extra demands that recruitment, and in some cases, data collection, placed on them. While every effort was made by researchers to support staff in this endeavour and to minimise the amount of work required, data collection was sporadic and considerable time and energy were invested in working with sites to improve this through email contact, site visits and the preparation and dissemination of site‐specific recruitment material.

Changes in personnel at some sites added further to these challenges through losses in momentum and the need for additional relationship building to establish understanding, trust and buy‐in from new staff. Best results were achieved when researcher efforts were complemented by a champion within the service willing to make data collection a priority, dedicate time and resources to it, and facilitate relationship building between researchers and peer staff. From our observations, consistency of involvement across co‐creation processes helped with the establishment of mutual goals and trust building, an opportunity that was lost when staff were only engaged in the latter stages of the project.

The careful alignment of research with program processes ensured that research was responsive to emergent needs and able to identify barriers or deficiencies in program implementation [1]. For example, over the course of the project, staff retention at some sites was identified as a pressing issue by members of the core team. Thus, strategies were implemented to engage current and former safe space staff in the research. These strategies included organising focus group discussions to capture collective experiences and perspectives, ensuring that these took place within work hours to maximise participation, and providing targeted opportunities for individual interviews with staff who had separated from the services. While health service managers from local health districts and nongovernment organisations involved in implementing the safe spaces partnered with researchers across the research cycle, safe space service managers, peer practice leads and peer workers played a more direct role in day‐to‐day implementation of both the safe space services, as well as the research, with implications for driving organisational and systemic change. To this end, the research took on increased significance for safe space staff by collecting data on their experiences of service implementation and the challenges faced by them.

Key partners' internal relationships and networks were pivotal to increasing research participation. These relationships and networks were cultivated to engage diverse health system stakeholders beyond the safe spaces to understand the positioning of the new services within local service landscapes. This was done by involving well‐positioned representatives within local health districts who enabled recruitment of emergency department, inpatient and community mental health staff to the research, and whose perspectives on how safe spaces fit within the broader health system were essential for understanding issues of implementation, effectiveness and sustainability. These and other professional networks were also leveraged to engage those involved in the co‐design of safe spaces in the research and to evaluate co‐design and implementation processes.

### 3.2 Co‐Evaluation

With co‐evaluation activities already established during co‐implementation stages via the embedding of data collection into routine practice, the boundaries between these two processes are somewhat blurred and porous. However, as Pearce and colleagues [4] note, co‐evaluation extends beyond data collection to include procedures for the co‐interpretation of findings. This is important because co‐creation approaches vary with regard to the level of end‐user input across different research phases, and claims of tokenism have been levelled at projects where lived experience involvement is limited to consultative rather than full participation [16, 17].

A unique feature of Co‐Creating Safe Spaces apart from being lived experience‐led and focused was its novel approach to embedding co‐creation into all aspects and stages of the research from conception through to interpretation, dissemination and translation of results. Planning and interpretation workshops and an inclusive authorship policy that invited all partners to lead or contribute to research papers aided this, providing an environment where co‐interpretation of study findings and their implications could effectively take place. This ensured that multiple perspectives were incorporated into evidence generation, bringing translation into practice much closer than the oft‐cited 17‐year research‐to‐practice gap [18].

At this point, we build upon the understanding of our co‐creation work to extend Pearce and colleagues' [4] construct of co‐evaluation by using a theory‐driven survey tool to operationalise and measure the health of our research collaborative. Given the increasing shift toward co‐creation in health, evidence of collaborative performance is important [19]. The core team was committed to evaluating ways of working to ensure that we were true to the principles agreed to at the outset [6]. Consistent with the project's strong lived experience leadership and involvement across all partner groups, 15 (75%) survey respondents identified as having a lived experience. To reduce individual identification of responses due to small numbers in some groups, findings were grouped as research partners 70% (*n* = 14) and service partners/lived experience representatives 40% (*n* = 8) with two members identifying as holding multiple roles.

#### 3.2.1 Co‐Evaluating Collaborative Structure

Table 1 reports on the structures that governed the collaboration. The results show that a majority of partners somewhat agreed or strongly agreed with statements relating to shared goals, shared resources, shared authority and shared accountability. Highest agreement was with statements relating to shared authority: ‘Partners are willing to distribute power to achieve our goals’ (*n* = 17 or 85%) and, shared accountability: ‘Partners feel ownership in the results/products of their work’ (*n* = 17 or 85%). These findings suggest that partners felt power was mutually shared and that the structures in place recognised and promoted shared ownership of the research.

**Table 1:** Collaborative Health Assessment Tool: Structure dimensions and descriptive statistics.

				Response *N* (%)					
	Statement	Partners^a^	*N*	Strongly disagree	Somewhat disagree	Undecided	Somewhat agree	Strongly agree	Don't know
Shared goal	Partners have a clear understanding of what a collaborative approach requires	All partners	20	1 (5)	4 (20)	—	10 (50)	5 (25)	—
		Research	13	1 (7.7)	4 (30.8)	—	7 (53.8)	1 (7.7)	—
		Service/Lived experience	7	—	—	—	3 (42.9)	4 (57.1)	—
Shared resources	The skills/expertise/specialisation that partners bring to the collaboration are appreciated	All partners	20	2 (10)	—	—	4 (20)	13 (65)	1 (5)
		Research	13	2 (15.4)	—	—	3.5 (26.9)	7.5 (57.7)	—
		Service/Lived experience	7	—	—	—	0.5 (7.1)	5.5 (78.6)	1 (14.3)
	We can access the data we need	All partners	20	1 (5)	3 (15)	3 (15)	9 (45)	2 (10)	2 (10)
		Research	13	1 (7.7)	3 (23.1)	2 (15.4)	5 (38.5)	1 (7.7)	1 (7.7)
		Service/Lived experience	7	—	—	1 (14.3)	4 (57.1)	1 (14.3)	1 (14.3)
Shared authority	Partners are willing to distribute power to achieve our goals	All partners	20	2 (10)	—	—	8 (40)	9 (45)	1 (5)
		Research	13	2 (15.4)	—	—	5 (38.5)	5 (38.5)	1 (7.7)
		Service/Lived experience	7	—	—	—	3 (42.9)	4 (57.1)	—
	All partners participate in decision‐making	All partners	20	—	5 (25)	1 (5)	7 (35)	7 (35)	—
		Research	13	—	3.5 (26.9)	1 (7.7)	6 (46.2)	2.5 (19.2)	—
		Service/Lived experience	7	—	1.5 (21.4)	—	1 (14.3)	4.5 (64.3)	—
Shared accountability	Each partner's areas of responsibility are clear and understood	All partners	20	—	4 (20)	3 (15)	8 (40)	5 (25)	—
		Research	13	—	4 (30.8)	3 (23.1)	5 (38.5)	1 (7.7)	—
		Service/Lived experience	7	—	—	—	3 (42.9)	4 (57.1)	—
	Partners feel ownership in the results/products of their work	All partners	20	—	3 (15)	—	8 (40)	9 (45)	—
		Research	13	—	3 (23.1)	—	5.5 (42.3)	4.5 (34.6)	—
		Service/Lived experience	7	—	—	—	2.5 (35.7)	4.5 (64.3)	—

^a^ Where > 1 role [i.e., research and service/lived experience partner] each role calculated at 0.5.

The statement relating to shared resources: ‘We can access the data we need’ resulted in an even spread of responses with just over half (*n* = 11 or 55%) somewhat or strongly agreeing with this statement, 20% (*n* = 4) somewhat or strongly disagreeing and 15% (*n* = 3) and 10% (*n* = 2) responding ‘undecided’ or ‘don't know’, respectively. The project generated a wide range of data (documentation, health administrative data, survey and interview data) and partners had different needs in terms of accessing and using it. This is evidenced by more than 30% (*n* = 4) of research partners somewhat or strongly disagreeing with this statement.

Further comparisons between partner groups showed consistent differences between research and service/lived experience partners. This was most pronounced in regard to shared goals and the statement: ‘Partners have a clear understanding of what a collaborative approach requires’: 38.5% (*n* = 5) of research partners somewhat or strongly disagreed with this statement, whereas 100% (*n* = 7) of service/lived experience partners somewhat or strongly agreed. Similar between‐group differences were evident with the statement on shared accountability: ‘Each partner's areas of responsibility are clear and understood’, which 30.8% (*n* = 4) of research partners somewhat disagreed with in comparison to 100% (*n* = 7) of service/lived experience partners whom somewhat or strongly agreed. One area where there was an overall alignment between groups was the statement: ‘All partners participate in decision‐making’, for which 25% (*n* = 5) of all participants (research and service/lived experience partners) somewhat disagreed.

#### 3.2.2 Co‐Evaluating Collaborative Process

Table 2 reports on the processes that shaped the collaboration. Statements relating to the CHAT dimensions whole‐system engagement: ‘Our collaboration has a diverse range of members’ and holding/authorising environment: ‘This collaboration has designed a safe environment in which disagreements and conflicts between members can be discussed’ received the highest percentage of agreement across all measures (*n* = 18 or 90%). This indicates that the majority of partners felt that sectoral diversity was accomplished by the inclusion of a broad range of health and community partners and that the project environment was responsive to partners' needs for safety and trust.

**Table 2:** Collaborative Health Assessment Tool: Process dimensions and descriptive statistics.

				Response *N* (%)					
	Statement	Partners^a^	*N*	Strongly disagree	Somewhat disagree	Undecided	Somewhat agree	Strongly agree	Don't know
Whole‐system engagement	Our collaboration has a diverse range of members (e.g. funders, local government reps, community members)	All partners	20	1 (5)	1 (5)	—	6 (30)	12 (60)	—
		Research	13	1 (7.7)	1 (7.7)	—	2 (15.4)	9 (69.2)	—
		Service/Lived experience	7	—	—	—	4 (57.1)	3 (42.9)	—
Communication flows	Communication among partners is effective (promotes understanding, cooperation and transfer of information)	All partners	20	1 (5)	4 (20)	—	9 (45)	6 (30)	—
		Research	13	1 (7.7)	3.5 (26.9)	—	7 (53.8)	1.5 (11.5)	—
		Service/Lived experience	7	—	0.5 (7.1)	—	2 (28.6)	4.5 (64.3)	—
Holding/authorising environment	This collaboration has designed a safe environment in which disagreements and conflicts between members can be discussed	All partners	20	2 (10)	—	—	6 (30)	12 (60)	—
		Research	13	2 (15.4)	—	—	5 (38.5)	6 (46.2)	—
		Service/Lived experience	7	—	—	—	1 (14.3)	6 (85.7)	—

^a^ Where > 1 role [i.e. research and service/lived experience partner] each role calculated at 0.5.

A small percentage of research partners (*n* = 2 or 15.4%) strongly disagreed with the statement: ‘This collaboration has designed a safe environment in which disagreements and conflicts between members can be discussed’. While the percentage was relatively low, it raised questions about safety, the types of conflict that manifested within the project, and how they were handled.

Given a diversity of project partners, communication flows are critical [13]. The majority of partners (*n* = 15 or 75%) agreed with the statement: ‘Communication among partners is effective’. However, there was a difference between research and service/lived experience partners with just over one‐third of research partners (*n* = 4.5 or 34.6%) disagreeing with this statement, compared with only 7% (*n* = 0.5) of service/lived experience partners.

#### 3.2.3 Co‐Evaluating Engagement and Decision‐Making Power

Table 3 reports aspects of engagement and decision‐making power drawn from the Trust and Empowerment Inventory [14]. The statement: ‘I feel I can influence outcomes important to me through our meetings’ received a high percentage of agreement overall (*n* = 16 or 80%). These results suggest a high level of satisfaction with meeting processes and the commitment and capacity of partners to contribute to shared outcomes. In contrast, the statement: ‘The other parties make good on their commitments’ resulted in an even spread of responses with 50% (*n* = 10) somewhat or strongly agreeing with this statement, 25% (*n* = 5) somewhat or strongly disagreeing, and 15% (*n* = 3) and 10% (*n* = 2) responding ‘undecided’ or ‘don't know’ respectively. A higher percentage of research partners somewhat or strongly disagreed with this statement (*n* = 4.5 or 34.6%) in comparison to service/lived experience partners who responded as ‘undecided’ or ‘don't know’ (*n* = 2.5 or 35.7%).

**Table 3:** Trust and empowerment inventory and descriptive statistics.

			Response *N* (%)					
Statement	Partners^a^	*N*	Strongly disagree	Somewhat disagree	Undecided	Somewhat agree	Strongly agree	Don't know
I feel I can influence outcomes important to me through our meetings	All partners	20	2 (10)	2 (10)	—	4 (20)	12 (60)	—
	Research	13	2 (15.4)	1 (7.7)	—	3 (23.1)	7 (53.8)	—
	Service/Lived experience	7	—	1 (14.3)	—	1 (14.3)	5 (71.4)	—
The other parties make good on their commitments	All partners	20	2 (10)	3 (15)	3 (15)	6 (30)	4 (20)	2 (10)
	Research	13	2 (15.4)	2.5 (19.2)	2 (15.4)	6 (46.2)	—	0.5 (3.8)
	Service/Lived experience	7	—	0.5 (7.1)	1 (14.3)	—	4 (57.1)	1.5 (21.4)

^a^ Where > 1 role [i.e. research and service/lived experience partner] each role calculated at 0.5.

## 4 Discussion

This study assessed processes of co‐implementation and co‐evaluation grounded in our own experiences from the Co‐Creating Safe Spaces project to better understand their complex and dynamic nature. It showed the importance of researchers and implementers working together across the entire implementation cycle. Building evaluation into co‐implementation within the project brought a number of benefits, most notably the responsiveness of research to local service needs that emerged during implementation. However, the demands of working in a busy crisis setting, with a diverse and often complex client base and within external funding constraints, impacted the level of safe space staff engagement in the research whereby implementers and researchers largely worked separately from each other [20]. This did not preclude individual sites from engaging in less formalised continuous quality improvement processes to improve professional practice, protocols and procedures. However, the complex funding, governance and management structures in which some safe spaces were nested meant that the use of evidence to drive service improvements at an organisational or systemic level during implementation was limited by decision‐making power being concentrated at higher management levels.

The engagement of stakeholders in earlier research stages is a common feature of the co‐creation literature, yet the input of implementers and specifically, recognition of co‐implementation as a process, is often overlooked within participatory health research discourses [15]. One of the strengths of Pearce and colleagues' [4] framework, therefore, is its ability to differentiate between diverse co‐creation activities. In describing the successes and challenges of effecting organisational and systemic change that reflect specific levels of researcher embeddedness and partnerships, our research highlights the importance of broader system changes to funding and governance structures to ensure research and service implementation are integrated in meaningful, sustained ways [15].

Findings from our research attest to the benefits of lived experience‐led and focused research and its capacity to establish the conditions necessary for sustaining equitable and healthy relationships among partners from different organisational cultures. However, the fostering of an environment conducive to collaborative discussion and decision‐making did not ensure that a safe environment in which disagreements and conflicts between members can be discussed was experienced by all. One such area of regular discussion and disagreement was language and framing. As in many fields, there are diverse views about language or engagement which may not have aligned with some research partners' preferences. The power imbalances that exist in the academic research space (within which this project ultimately resided) may also have buttressed some partners from criticism and created a space where disagreement or conflict could neither be created nor resolved.

Findings related to an individual partner's areas of responsibility and ability to deliver on commitments were closely linked. With individual partners having different stakes in the project, these findings may simply reflect varying levels of involvement, with those whose time commitments were significantly less expressing greater satisfaction. For a small number of research partners, many of whom were involved with the day‐to‐day running of the project, there were times when they felt other research and service/lived experience partners did not deliver on responsibilities set out in the funding proposal and MIA.

The findings provide valuable insights into governance and management systems and their influence on how co‐creation occurs between university, health and community partners [15]. The establishment of an MIA between partners, for example, did not assure that responsibility and accountability for actions relating to shared decisions were understood and acted upon. Indeed, its ineffectuality in relation to timely research governance processes, including open data sharing with some health partners, indicated that rather than innovating structurally to create new collaborative or partnership models, our collaboration reflected and reproduced existing hierarchies and power relations [21]. To some extent, these mirror findings around power structures we observed within grant‐funded university research and the limitations of power devolution. Due to the project being grant‐funded research, it was necessarily located within academic structures and hierarchies. Despite a clear decision by the lead investigator to devolve power and responsibility, governance structures did not always support democratic decision‐making. This description of the hierarchies and power relationships within local partnerships reveals the contradictory and complex reality of collaboration between health, community and university sectors and the counterproductive governance and management systems that frequently inhibit and impede co‐creation research [21].

Given that co‐creation in research is part of a wider politics of knowledge in which multiple imperatives, interests and power relations co‐exist, research that reports on the micro‐dynamics of local collaborative practices is important [21]. Co‐created research, particularly in the field of mental health and suicide prevention, is frequently pursued not necessarily because it is effective and equitable, but because of external pressures based on the assumption that it is [19]. As a result, considerable investments are made to establish collaborative networks without addressing the issue of collaboration effectiveness [19].

Yet evidence of effectiveness, or for that matter, how best to measure effectiveness and for whom collaboration should be most beneficial, is sparse and subject to ongoing debate [19]. Co‐creation frameworks or standardised definitions such as those proposed by Pearce et al. [4] are useful for thinking about what constitutes co‐creation. However, they do little to draw attention to the complexities, interdependencies and dynamics of collaborative environments [19]. In evaluating collaborative practices within the Co‐Creating Safe Spaces project through critical reflection and a theory‐driven evaluation tool, this article contributes new insights into participation, collaboration and co‐creation within local partnerships. Evaluation strategies that focus on how well partners work together across interacting domains are a key missing piece of co‐evaluation as a concept and practice, and their implementation is essential to furthering the practice of co‐created research [22].

## 5 Conclusion

The Co‐Creating Safe Spaces project was bold in both its aim and its ongoing commitment to research co‐creation. Despite the effect on project scope and outcomes created by challenges that were unable to be resolved, such as health departments not providing agreed data, overall, the collaboration was viewed positively, and the project produced good early evidence on safe space implementation and effectiveness. We found co‐creation to be a fluid process that is difficult to compartmentalise into stages and still influenced by the hierarchical systems within which it was being enacted. Our project demonstrates that co‐creation success depends on constant attention to relational work coupled with ongoing and honest reflexive practice to identify and address challenges.

## Author Contributions

**Scott J. Fitzpatrick:** conceptualisation, investigation, methodology, formal analysis, project administration, data curation, supervision, writing–original draft, writing–review and editing, visualisation. **Heather Lamb:** investigation, funding acquisition, writing–original draft, formal analysis, data curation, conceptualisation. **Erin Oldman:** writing–original draft, formal analysis, investigation, conceptualisation. **Melanie Giugni:** conceptualisation, investigation, writing–original draft, formal analysis, data curation, software. **Cassandra Chakouch:** investigation, writing–original draft, formal analysis, conceptualisation. **Alyssa R. Morse:** writing–original draft, conceptualisation, funding acquisition, investigation. **Amelia Gulliver:** writing–original draft, funding acquisition, visualisation, investigation. **Erin Stewart:** writing–original draft, investigation. **Helen T. Oni:** writing–original draft, investigation. **Benn Miller:** writing–original draft, investigation. **Bronwen Edwards:** writing–original draft, investigation. **Kelly Stewart:** writing–original draft, investigation. **Vida Bliokas:** writing–original draft, funding acquisition, investigation. **Louise A. Ellis:** funding acquisition, writing–original draft, investigation. **Fiona Shand:** funding acquisition, writing–original draft, investigation. **Alison L. Calear:** funding acquisition, writing–original draft, investigation. **Michelle Banfield:** conceptualisation, investigation, funding acquisition, writing–original draft, writing–review and editing, formal analysis, project administration, data curation, supervision, resources, methodology.

## Conflicts of Interest

The authors declare no conflicts of interest.

## Data Availability

The authors confirm that the data supporting the findings of this study are available within the article or its supplementary materials.
